# Concordance and diagnostic accuracy of vasodilator stress cardiac MRI and 320-detector row coronary CTA

**DOI:** 10.1007/s10554-013-0300-0

**Published:** 2013-10-12

**Authors:** Marcus Y. Chen, W. Patricia Bandettini, Sujata M. Shanbhag, Sujethra Vasu, Oscar J. Booker, Steve W. Leung, Joel R. Wilson, Peter Kellman, Li-Yueh Hsu, Robert J. Lederman, Andrew E. Arai

**Affiliations:** Advanced Cardiovascular Imaging Laboratory, Division of Intramural Research, Department of Health and Human Services, Cardiovascular and Pulmonary Branch, National Heart Lung and Blood Institute, National Institutes of Health, 10 Center Drive, Building 10, Room B1D416, Bethesda, MD 20892-1061 USA

**Keywords:** Coronary artery disease, Cardiac magnetic resonance imaging, Cardiac computed tomography, Myocardial perfusion imaging

## Abstract

Vasodilator stress cardiac magnetic resonance (CMR) detects ischemia whereas coronary CT angiography (CTA) detects atherosclerosis. The purpose of this study was to determine concordance and accuracy of vasodilator stress CMR and coronary CTA in the same subjects. We studied 151 consecutive subjects referred to detect or exclude suspected obstructive coronary artery disease (CAD) in patients without known disease or recurrent stenosis or ischemia in patients with previously treated CAD. Vasodilator stress CMR was performed on a 1.5 T scanner. CTA was performed on a 320-detector row system. Subjects were followed for cardiovascular events and downstream diagnostic testing. Subjects averaged 56 ± 12 years (60 % male), and 62 % had intermediate pre-test probability for obstructive CAD. Follow-up averaged 450 ± 115 days and was 100 % complete. CMR and CTA agreed in 92 % of cases (κ 0.81, *p* < 0.001). The event-free survival was 97 % for non-ischemic and 39 % for ischemic CMR (*p* < 0.0001). The event-free survival was 99 % for non-obstructive and 36 % for obstructive CTA (*p* < 0.0001). Using a reference standard including quantitative invasive angiography or major cardiovascular events, CMR and CTA had respective sensitivities of 93 and 98 %; specificities of 96 and 96 %; positive predictive values of 91 and 91 %; negative predictive values of 97 and 99 %; and accuracies of 95 and 97 %. Non-ischemic vasodilator stress CMR or non-obstructive coronary CTA were highly concordant and each confer an excellent prognosis. CMR and CTA are both accurate for assessment of obstructive CAD in a predominantly intermediate risk population.

## Introduction

Over 9 million stress tests [1] and 1 million diagnostic invasive angiograms [2] are performed annually in the United States. Thus the typical patient undergoing stress testing is substantially different than the typical patient undergoing invasive angiography. In a recent controversy, an abnormal stress test has limited value for determining obstructive coronary artery disease (CAD) on invasive angiography [3]. However, others conclude those results are predictable based on referral bias and the shortcomings of the reference standard [4].

Multi-slice cardiac CT angiography (CTA) [5] and vasodilator stress cardiac MRI (CMR) [6] are two emerging techniques to assess for obstructive CAD. The concordance and relative diagnostic accuracy of these two different non-invasive imaging modalities is not established.

The specific aim of this study was to evaluate the concordance and diagnostic accuracy of 320-detector row coronary CTA and vasodilator CMR for detection of obstructive CAD. We recognized that the majority of intermediate risk patients would not undergo coronary angiography. Thus, diagnostic accuracy of the newer tests was assessed using subsequent diagnostic tests, revascularization, or major cardiac events. Findings from invasive angiography combined with patient outcomes were then used as a reference standard for determining the diagnostic accuracy. We hypothesized that (1) CTA and CMR are accurate for the assessment of obstructive CAD and (2) each modality provides good discrimination for future cardiac events.

## Methods

### Study design

This study was performed at a single referral center where subjects who underwent both CMR and CTA were identified from Clinical Trial Registration NCT00027170 URL: http://www.clinicaltrials.gov. Data was prospectively acquired and retrospectively analyzed. Invasive catheterization was recommended for subjects with either an abnormal or positive CMR or CTA. Subjects were followed for downstream diagnostic procedures such as invasive angiography or cardiovascular events. The institutional review board approved the study, and all subjects consented in writing.

### Study cohort

Eligible subjects were at least 18 years old, and referred for non-invasive testing to (a) detect or exclude suspected obstructive CAD in patients with no known disease, or (b) assess for possible recurrent stenosis or ischemia in patients with previously completely revascularized and treated CAD. Subjects were excluded if they were pregnant, ineligible for CMR (cerebral aneurysm clips, metal shrapnel or implanted metallic devices, etc.), or if estimated glomerular filtration rate was <30 mL/min/1.73 m^2^ body surface area [7]. The pre-test likelihood of CAD was estimated according to criteria developed by Diamond and Forrester [8].

### Stress cardiac MRI

CMR was performed at 1.5 T (Avanto or Espree, Siemens, Erlangen, Germany) using a 32 channel surface coil. A first pass bolus of 0.05 mmol/kg Gd-DTPA (gadopentetate dimeglumine, Bayer Healthcare Pharmaceuticals, Wayne, NJ, USA) was administered 70 s after regadenoson 0.4 mg intravenous bolus or 4 min after completing an intravenous infusion of dipyridamole 0.56 mg/kg administration over 4 min. First-pass perfusion was imaged using steady state free precession MRI of three left ventricular short-axis slice locations (base, mid and apex), after which aminophylline 100–200 mg was administered to reverse the vasodilator agent. Typical imaging parameters included a saturation preparation pulse, readout excitation flip angle 50°, repetition time (TR) 2.3 ms, echo time (TE) 1.1 ms, bandwidth 1,085 Hz/pixel, acquisition matrix 128 × 80, field of view (FOV) 360 × 270 mm, slice thickness 8 mm, and temporal resolution 92 ms with parallel imaging acceleration factor of 2. Next, cine images of cardiac function were obtained, followed by baseline (“rest”) first pass perfusion using an additional 0.05 mmol/kg Gd-DTPA at least 20 min after stress imaging.

Readers were blinded to other test results during interpretation on a dedicated workstation (Leonardo, Siemens, Erlangen, Germany) and images were interpreted visually. Abnormal studies were defined as stress-induced perfusion defects of any size that were more severe than at rest, and similar defects on both stress and rest images were considered an artifact. Late gadolinium enhanced images did not influence the reading of perfusion scans.

### Cardiac CT

ECG-gated CTA was performed in an axial fashion on a 320-row scanner (Aquilion ONE, Toshiba, Japan) with a gantry rotation time of 0.35 s. Oral and intravenous metoprolol or diltiazem (if beta blockers were contraindicated) was administered to achieve a target resting heart rate <60 beats/min. Nitroglycerin vasodilated CTA images were acquired after intermittent bolus tracking of iopamidol-370 (Bracco Diagnostics, Princeton, NJ, USA) radiocontrast (1–1.5 mL/kg) in the descending aorta using a trigger threshold of 180 Hounsfield units. To minimize radiation exposure, tube voltage and current were adjusted to body size and volumetric acquisition in an axial manner with prospective electrocardiogram triggered imaging was used when possible [9, 10]. Images were reconstructed with 0.5 mm slice thickness and 0.25 mm increment using kernel FC03. Readers were blinded to results of all other testing and used a dedicated workstation (VitreaFX, Vital Images, Minnetonka, MN, USA). Interpretations followed published guidelines [11]. Examinations were prospectively considered positive for obstructive CAD if there was a ≥50 % stenosis in a coronary artery diameter ≥1.5 mm to maintain sensitivity for detecting obstructive CAD due to known limitations in spatial and temporal resolution for CTA [5]. Radiation dose was estimated using a dose–length product conversion factor of 0.014 mSv/(mGy × cm) [9].

### Invasive angiography

Catheter-based angiography was recommended if either or both CMR or CTA were positive, and the referring physician made the clinical determination on how to proceed. Obstructive CAD was defined as a stenosis ≥70 % by quantitative coronary angiography (CAAS II QCA, Pie Medical Imaging, Maastricht, The Netherlands) of the most severe stenosis per vessel. Quantification was performed blinded to results of all other testing.

### Follow-up

Outcome data for major cardiovascular events (death, non-fatal myocardial infarction, coronary revascularization, stroke, admission for unstable angina) or subsequent testing were obtained from a standardized questionnaire based on telephone interviews or written responses. Any clinical events were confirmed after review of outpatient or hospital medical records. Only the first of multiple events was considered for analysis.

### Statistical analysis

We compared assessments of diagnostic accuracy of stress CMR and CTA using a combined reference standard of clinical outcome on follow-up or quantitative invasive catheterization. Data were analyzed on a per patient basis. Statistical analysis was performed using SPSS version 17 (IBM, Somers, NY, USA). Data are presented as mean ± standard deviation if normally distributed. Otherwise, descriptive parameters are presented as median and interquartile range. Confidence intervals are reported at the 95 % limits. Concordance was assessed with a κ statistic. Survival distributions for the time to event were estimated using the Kaplan–Meier method. The differences between survival distributions were assessed using the log-rank test. Statistical significance was defined as *p* < 0.05. Differences in receiver operator curves were evaluated using the univariate *z* score test where two-tailed *p* < 0.05 values were considered to be statistically significant.

## Results

### Demographics

All 151 subjects completed both CTA and CMR exams between February 2009 and June 2010. The median time between examinations was 0 days (interquartile range 0–8 days) because 90 (60 %) exams were performed on the same day. Table 1 describes the clinical characteristics of subjects in this study. The median age was 56 years (interquartile range, 48–63) and 60 % were male. A majority of subjects had no known CAD (88 %), intermediate pre-test probability CAD (62 %), hypertension (70 %), hyperlipidemia (70 %), and were taking cardiovascular medications (82 %). The prevalence of overweight or obese subjects (76 % with a body mass index ≥25 kg/m^2^) was similar to the general United States population [12]. The median estimated radiation dose from CTA was 4.9 mSv (interquartile range 3.2–6.7 mSv).

**Table 1:** Baseline characteristics of the 151 subjects

Characteristics	n = 151
Age (years)	
Median	56
Interquartile range	48–63
Maximum, minimum	20, 83
Male sex—n (%)	91 (60 %)
Race—n (%)	
White	95 (63 %)
African American	26 (17 %)
Asian	27 (18 %)
More than one race	1 (1 %)
Native Hawaiian or Pacific islander	2 (1 %)
Ethnicity—n (%)	
Non-hispanic	145 (96 %)
Hispanic	6 (4 %)
Body mass index	
Median	27
Interquartile range	25–32
Normal <25	36 (24 %)
Overweight 25–30	67 (44 %)
Obese 30–40	38 (25 %)
Morbid obesity >40	11 (7 %)
CAD risk factors—n (%)	
Family history	46 (30 %)
Hypertension	106 (70 %)
Dyslipidemia	105 (70 %)
Diabetes	30 (20 %)
Smoking	44 (29 %)
Known CAD—n (%)	17 (11 %)
Percutaneous coronary intervention	13 (9 %)
Coronary artery bypass surgery	4 (3 %)
No prior CAD—n (%)	134 (89 %)
Pretest probability for CAD	
Low	38 (29 %)
Intermediate	83 (62 %)
High	12 (9 %)
Symptoms	
Typical angina	37 (25 %)
Atypical angina	64 (42 %)
Non-anginal chest pain	24 (16 %)
Asymptomatic with prior equivocal stress test	21 (14 %)
Asymptomatic	4 (3 %)
Asymptomatic with new onset congestive heart failure	1 (1 %)
Medications—n (%)	
Anti-platelet/anti-coagulant medications	
Aspirin	78 (52 %)
Clopidogrel	9 (6 %)
Warfarin	4 (3 %)
Anti-hypertensive medications	
Beta blocker	64 (42 %)
Calcium channel blocker	20 (13 %)
ACE inhibitor	44 (29 %)
ARB	20 (13 %)
Diuretic	39 (26 %)
Long acting nitrates	13 (9 %)
Other anti-hypertensive (clonidine, renin inhibitor)	3 (2 %)
Lipid medications	
Statin	83 (55 %)
Other lipid therapy	32 (21 %)
Diabetes medications	
Insulin	4 (3 %)
Oral agents	25 (17 %)
No cardiovascular medications	27 (18 %)
Lab testing	
eGFR (mL/min)	
Median	79
Interquartile range	67–92
Left ventricular ejection fraction	
Median	62 %
Interquartile range	57–68 %
Agatston coronary calcium score	
≥1,000	12 (8 %)
400–999	17 (11 %)
100–399	26 (17 %)
10–99	25 (17 %)
1–9	9 (6 %)
0	62 (41 %)

*CAD* coronary artery disease, *ACE* angiotensin converting enzyme, *ARB* angiotensin II receptor blocker, *eGFR* estimated glomerular filtration rate

### Prevalence of CAD

By any assessment, the prevalence of obstructive CAD was typical of non-invasive stress test populations: CTA 30 % (45 of 151), CMR 28 % (43 of 151), invasive angiography 24 % (36 of 151 overall but only 43 underwent). The overall severity of obstructive CAD detected by CTA was single vessel 15 % (23 of 151), two-vessel 11 % (26 of 151) and three-vessel 4 % (6 of 151). Since the number of subjects who underwent invasive angiography was lower (n = 43), the prevalence of obstructive CAD was higher but with a similar distribution: single vessel 44 % (19 of 43), two-vessel 26 % (11 of 43) and three-vessel 14 % (6 of 43). None had significant left main disease by either CTA or invasive angiography.

### Concordance of CMR stress perfusion and CTA

**Fig. 1:**
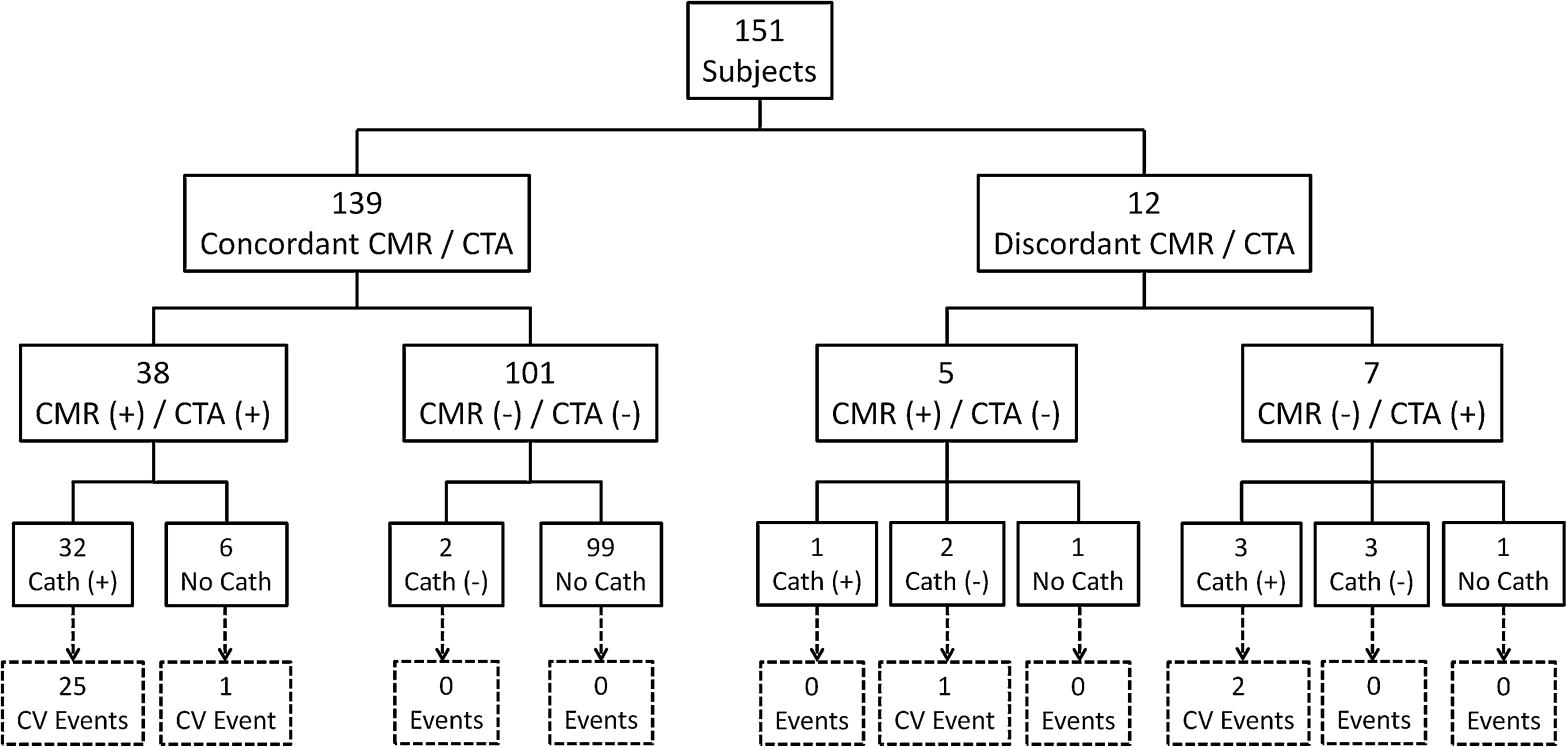
Results of invasive coronary catheterization and cardiovascular events in patients with concordant or discordant CMR and CTA. CMR was concordant with catheterization in 88.4 % (38 of 43) and CTA was concordant with catheterization in 90.7 % (39 of 43) of patients. Overall, 26 of 29 events occurred in patients with a positive CMR and CTA. Of the 108 negative CMRs, there were 2 cardiovascular events (revascularization). Of the 106 negative CTAs, there was 1 cardiovascular event (non-fatal myocardial infarction and subsequent percutaneous coronary intervention) from progression of non-obstructive coronary artery disease. This patient had invasive angiography following CMR and CTA, and again at the time of the myocardial infarction

Not considering any other data, 139 subjects (92 %) had concordant CMR and CTA findings and 12 had discordant findings (Fig. 1). The κ value of 0.81 (*p* < 0.001) is a level generally considered excellent. There were 38 subjects who had concordant positive findings and 101 who had concordant negative findings. The discordant cases included 7 subjects with a positive CTA and negative CMR, and 5 subjects with a negative CTA and a positive CMR. Table 2 shows the two-by-two contingency table. The proportion of discordant results did not indicate a significant difference between CMR and CTA (McNemar test, *p* = 0.77).

**Table 2:** Concordance of CMR and CTA findings is demonstrated on the contingency table of CMR and CTA abnormal or normal findings

	CTA (+)	CTA (−)
CMR (+)	38	5
CMR (−)	7	101

There is strong agreement (92 %) and correlation (κ value 0.81, *p* < 0.001) between modalities

### Follow-up

Subjects were surveyed to ensure that no clinically significant CAD was missed by CTA or CMR. Follow-up duration averaged 450 ± 115 days (inter-quartile range 376–512 days) and was obtained in 100 % of subjects. Overall, 43 subjects (30 %) had invasive angiography and 29 major cardiac events occurred (1 death, 2 non-fatal myocardial infarctions, 11 percutaneous interventions, and 15 coronary artery bypass surgeries). One death and one non-fatal myocardial infarction occurred in two separate subjects who each had a concordant positive CTA and positive CMR. An additional non-fatal myocardial infarction occurred 8 months after testing in a subject who had non-obstructive CAD on CTA, ischemia on CMR, and non-obstructive CAD on intial invasive catheterization. Subsequent invasive angiography at the time of the myocardial infarction demonstrated progression of CAD and a percutaneous coronary intervention was performed. There were no strokes. Overall, two subjects were hospitalized for non-fatal myocardial infarction and no subjects were hospitalized for unstable angina.

During the follow-up period, 6 subjects had clinically-driven non-invasive diagnostic testing performed and the findings were not different from the initial CMR or CTA findings. In 2 subjects who had ischemia on CMR, obstructive CAD on CTA, and obstructive CAD on invasive angiography without revascularization, the subsequent nuclear perfusion scintigraphy confirmed CMR or CTA results. Similarly, 4 subjects with no ischemia on CMR and non-obstructive CAD on CTA had further non-invasive testing (3 nuclear perfusion scintigraphy and one stress echocardiogram) that were all negative.

**Fig. 2:**
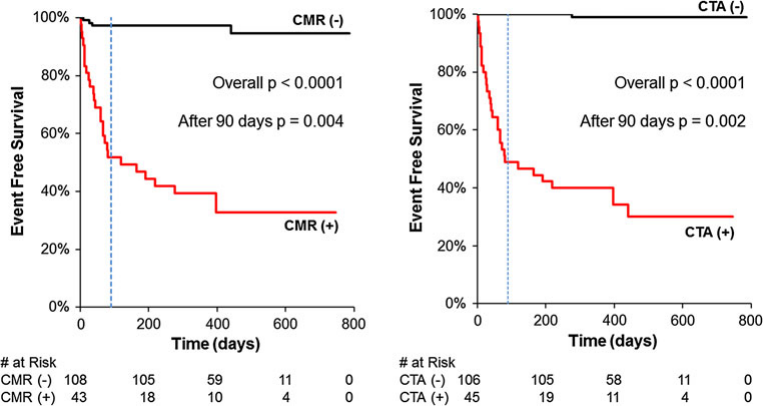
Kaplan–Meier survival distributions for death, non-fatal myocardial infarction or coronary revascularization based on presence or absence of ischemia on cardiac magnetic resonance (CMR) or obstructive coronary artery disease on cardiovascular CT (CTA). Very few events occurred in either the negative CMR or negative CTA groups. Overall, the separation is statistically significant for each curve (*p* < 0.001 by log-rank test). Many cardiovascular events occurred within 90 days (*dotted line*); however the separation between the two groups continues beyond 90 days (*p* = 0.004 for CMR, *p* = 0.002 for CTA)

**Fig. 3:**
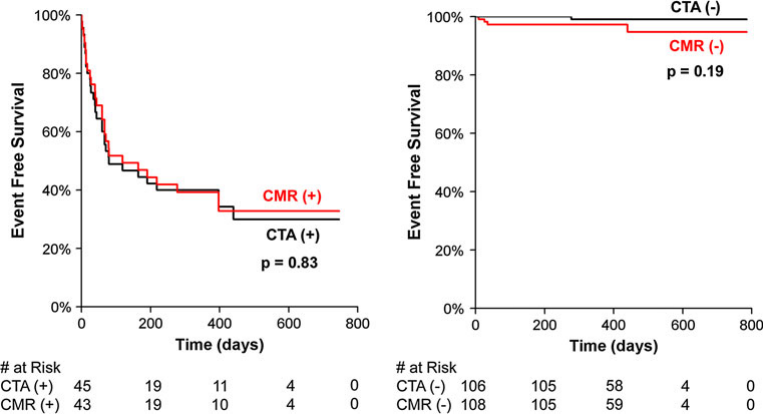
Kaplan–Meier *curves* for the comparison of abnormal and normal results of CMR and CTA findings. Differences between *curves* are statistically not significant (*p* = 0.83 and 0.19 by log-rank test, respectively)

Figures 2 and 3 illustrate the time to Kaplan–Meier event distributions for subjects based on CTA and CMR findings. Based on the 29 major cardiac events, the 450 day event free survival was 97 % for non-ischemic and 39 % for ischemic findings on CMR (*p* < 0.0001) and 99 % for non-obstructive and 36 % for obstructive CAD on CTA (*p* < 0.0001). Neither test missed the one subject that died.

### Diagnostic accuracy of CMR stress perfusion and CTA determined by catheterization and follow-up data

**Fig. 4:**
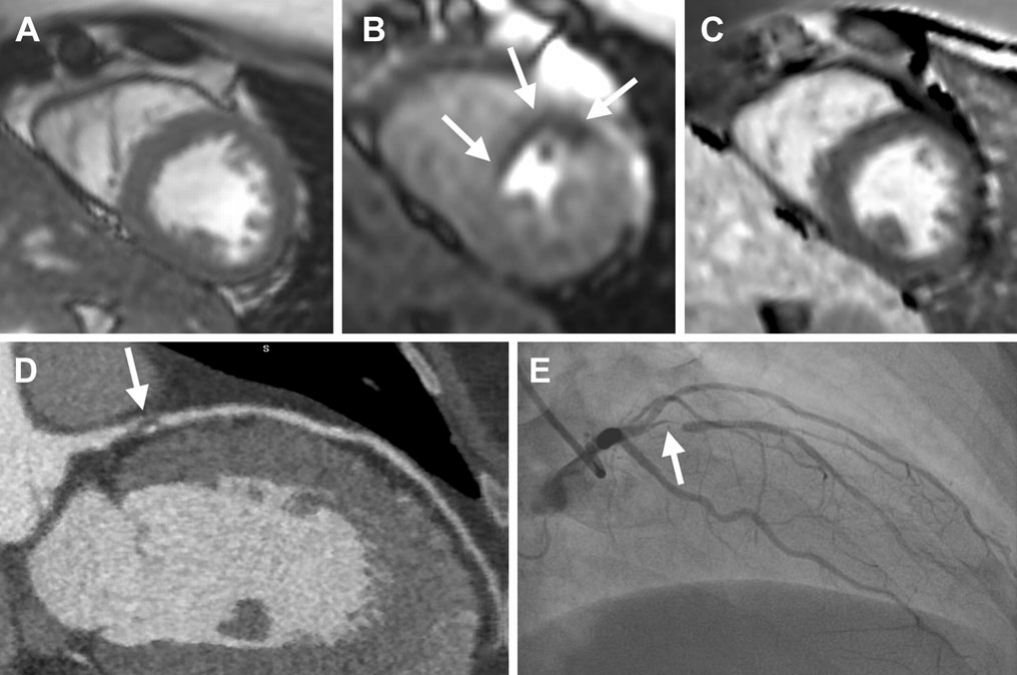
Example of concordant positive findings on CMR and CTA from a 46 year old female with no prior cardiovascular history presenting with intermediate pre-test probability for CAD and an equivocal nuclear SPECT study. The short axis cine CMR showed normal wall thickness (**a**), an anterior and anteroseptal stress induced perfusion defect (**b**, *arrows*) without evidence of myocardial infarction on late gadolinium enhancement imaging (**c**). The CTA showed an obstructive mixed calcified and non-calcified stenosis of the proximal left anterior descending (LAD) coronary artery (**d**, *arrow*). Invasive angiography confirmed a severe proximal stenosis of the LAD (**e**, *arrow*)

Thirty-eight subjects had concordant positive results on CTA and CMR (Fig. 4). Of these, 32 (84 %) underwent invasive catheterization, which confirmed significant CAD in all. Of the remaining 6 subjects with concordant positive CTA and CMR, 4 were advised to undergo catheterization but declined. The primary cardiologist did not proceed with invasive angiography for the remaining 2 subjects due to concern for malignancy in one patient and known CAD in the other patient.

One-hundred and one subjects had concordant negative results on CTA and CMR. Of these, two underwent invasive catheterization and both tests were negative.

**Fig. 5:**
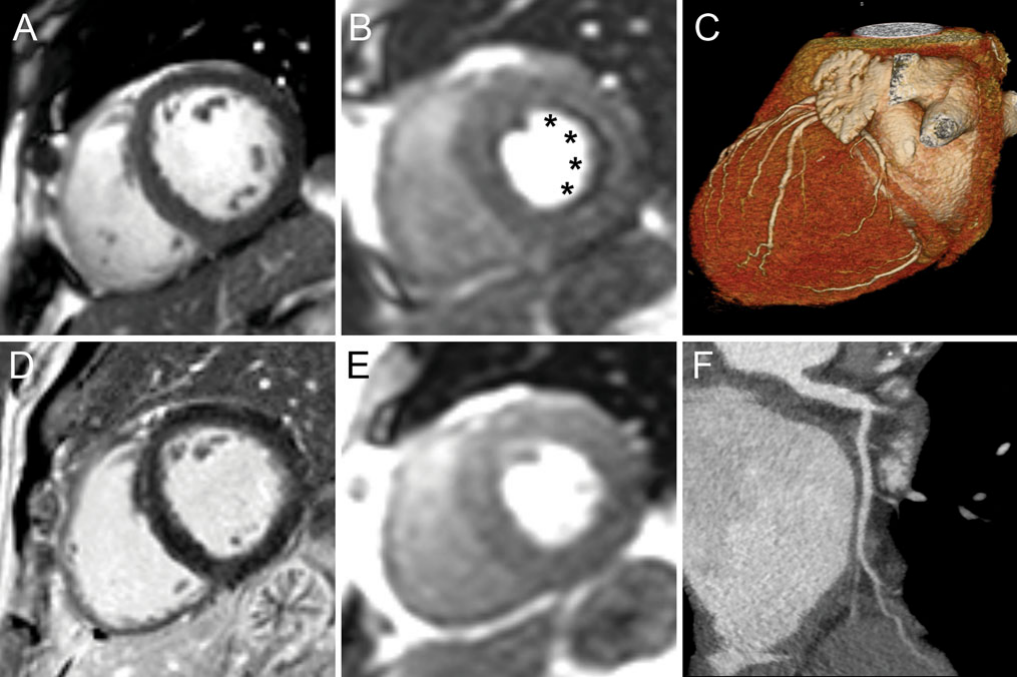
Example of a false positive CMR determined by prognosis in a 65 year old male with no prior cardiovascular history and intermediate pre-test probability for CAD and non-obstructive CAD on CTA. Short-axis cine CMR (**a**), subendocardial vasodilator stress induced perfusion defect involving the lateral and inferolateral walls (*asterisks*, **b**) which are not present on rest imaging (**e**). Delayed enhancement imaging is normal without evidence of myocardial infarction (**f**). Coronary CTA 3D surface (**c**) shows normal distribution of vessels to the lateral wall and non-obstructive CAD disease of the left main and left circumflex (**f**)

**Fig. 6:**
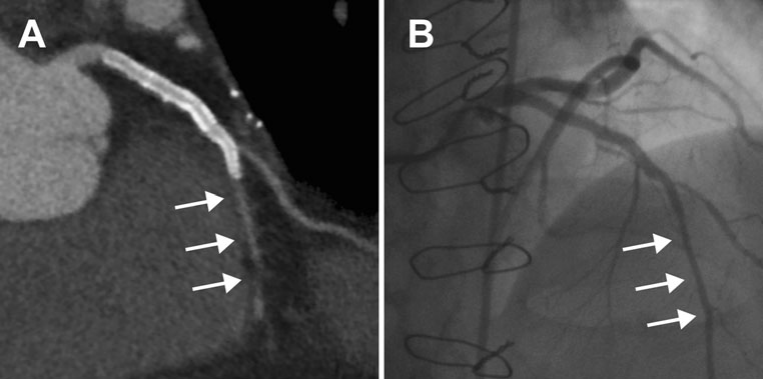
Example of a false positive CTA from a 54 year old male with atypical chest pain, known CAD treated with single vessel bypass surgery to the left anterior descending (LAD) followed a proximal LAD stent (3.0 mm diameter) 6 years later due to graft occlusion. The CTA had poor contrast opacification of the LAD (**a**, *arrows*) beyond the stent which was interpreted as a severe stenosis. This finding was confirmed on axial and multi-planar reformat imaging. Invasive angiography demonstrated a patent proximal LAD stent with good opacification of the distal vessel (**b**, *arrows*). CMR demonstrated normal stress perfusion and no evidence of myocardial infarction (not shown)

Twelve subjects had discordant results on CMR and CTA (see Figs. 5, 6 for examples). All were advised to undergo invasive catheterization, and 9 (75 %) complied. There were 3 false positive CTAs and 1 false negative CTA. There were 3 false negative CMRs and 2 false positive CMRs. The three subjects who declined invasive angiography were classified based on outcome information (no cardiovascular events yielding 2 false positive CMR and one false positive CTA).

Table 3 summarizes the diagnostic accuracy analysis using a reference standard composed of a composite of invasive angiography (QCA ≥70 %) and outcomes (death, myocardial infarction and revascularization). For CMR, the sensitivity was 93 %, specificity 96 %, positive predictive value 91 %, negative predictive value 97 %, and accuracy 95 %. For CTA, the sensitivity was 98 %, specificity 96 %, positive predictive value 91 %, negative predictive value 99 %, and accuracy 97 %.

**Table 3:** Diagnostic accuracy of CMR and CTA for the detection of obstructive CAD using a composite reference standard of invasive angiography (QCA 70 %) and patient outcomes (death, myocardial infarction and revascularization)

Diagnostic Accuracy of CMR		Diagnostic Accuracy of CTA	
Sensitivity	93 (83–98)	Sensitivity	98 (89–100)
Specificity	96 (93–98)	Specificity	96 (93–97)
PPV	91 (81–96)	PPV	91 (83–93)
NPV	97 (94–99)	NPV	99 (96–100)
Accuracy	95 (90–98)	Accuracy	97 (92–98)

	Composite (+)	Composite (−)		Composite (+)	Composite (−)
CMR (+)	39	4	CTA (+)	41	4
CMR (−)	3	105	CTA (−)	1	105

Values in parentheses represent 95 % confidence intervals. Matching contingency tables are shown underneath. Consistent with the purpose of using outcomes to ensure that CTA or CMR did not miss important CAD, there were 3 outcomes beyond the 42 cases with invasive angiography confirmation. Two of three events were detected by CTA leading to an overall sensitivity of 99 %. CMR detected all 3 outcomes with an overall sensitivity of 93 %

*PPV* positive predictive value, *NPV* negative predictive value

Receiver operating curve analyses demonstrates no statistically significant difference (*p* = 0.40) between CMR (area under curve 0.873, 95 % CI 0.797–0.949) and CTA (area under curve 0.921, confidence interval 0.870–0.972) for predicting future cardiovascular events.

## Discussion

Our study represents the first evaluation of concordance and accuracy using 320-row CTA and vasodilator stress CMR in the same subjects. The agreement for evaluating obstructive CAD between vasodilator CMR and CTA is excellent (agreement 92 %, κ value 0.81). Both tests are accurate for the assessment of obstructive CAD in a predominately intermediate risk population.

Myocardial perfusion imaging by CMR or nuclear techniques assesses the physiological significance of CAD, whereas CTA provides anatomic visualization of the location and severity of atherosclerosis. The inherent differences in assessing physiology versus anatomy have been used to explain discordant findings between CTA and both SPECT and PET imaging [13]. However, in this study, the correlation between CTA and CMR was excellent (agreement 92 %, κ 0.81) which is consistent with another smaller study [14]. CMR appears to have higher diagnostic accuracy for detecting CAD than SPECT [15]. When evaluating two identical physiological tests (nuclear SPECT) for the detection of ischemia performed using the same vasodilator (adenosine) in two different settings, the agreement is moderate at 62 % with a κ value of 0.46[16]. Simultaneous evaluation for two different imaging modalities (echocardiography and nuclear SPECT) during the same dobutamine stress evaluation have demonstrated an agreement of 69 % with a κ value of 0.25[17]. Thus, the agreement between CTA and CMR in the current study is excellent compared with the concordance of conventional tests.

The diagnostic accuracy of CTA has been evaluated using meta-analyses [18]; however, the studies did not include 320-row CT scanners. The existing small single center 320-row CT diagnostic accuracy trials [19–21] studied a high pre-test probability for CAD in patients scheduled for invasive angiography. Enrolling subjects who are destined for reference standard test of invasive angiography introduces pre-test referral bias by enriching the cohort with high prevalence disease subjects. This bias generates more true positive results and potentially exaggerates test sensitivity, as seen in the CTA validation literature [5]. Due to the small but serious risks of invasive angiography, ethical considerations preclude performing widespread invasive angiography in research subjects with a low to intermediate prevalence of CAD, as in this study. Thus the nearly 2 year outcome data in the current study is valuable for insuring that no significant disease was missed. Lack of revascularization, myocardial infarction or death over 2 years after a test is consistent with excellent sensitivity.

Similarly, CMR meta-analyses [22] have been performed; however, the amount of gadolinium contrast, pulse sequence utilized and interpretation method varied between studies. Similar to CTA meta-analyses, many of the CMR diagnostic accuracy trials studied populations with high pre-test probability of CAD destined for invasive angiography and thus do not reflect typical populations or real-world practice. The current study demographics are typical of patients referred for stress test to evaluate for obstructive CAD.

Our work demonstrates that either an anatomic based (CTA) or physiologic assessment (CMR) are accurate for the detection of obstructive CAD. Due to escalating medical costs, it is not necessary to perform both of these two complementary non-invasive tests to diagnose CAD.

The prognostic value of 320-row CT in the current study is comparable to a recent meta-analysis [23] and the sample size of the current trial would fit within the middle third of the sample sizes included within the meta-analysis. The strength of our study is that the predominately intermediate risk group of patients in this cohort reflects a typical outpatient population, and therefore the prognostic information is broadly applicable. In addition, there was 100 % follow-up achieved evaluating both hard (death and non-fatal myocardial infarction) and soft (revascularization and hospitalization) events.

CMR in one exam can provide more than an assessment for ischemia because it offers a comprehensive evaluation of cardiac anatomy, function, valve disease, tissue characterization, viability and fibrosis assessment [24] in one setting. However, some patients with metallic implants or claustrophobia are ineligible for CMR exams. CTA is more widely available than CMR, and exam times are shorter. Due to the concerns over the biologic effects of ionizing radiation [25], coronary CTA evaluations are constrained to a very limited portion of the cardiac cycle [9] to evaluate coronary anatomy.

### Limitations

Quantitative coronary angiography has known limitations as a reference standard, because it does not necessarily incorporate lesion characteristics such as length, shape, eccentricity, collateral blood flow, or vasomotor tone and therefore may misrepresent the physiological significance of lesions, especially of intermediate severity [26]. We did not systematically confirm lesion functional severity in this study using fractional flow reserve during catheterization; however, these measurements are not routinely practiced [27] and could not have been performed in the 72 % of subjects who did not have invasive coronary angiography. Stress CMR has been validated against fractional flow reserve for the detection of significant CAD [28]. A limited number of subjects in this study with abnormal CMR or CTA elected not to undergo invasive angiography, but rather chose medical therapy [29]. CMR and CTA were used as diagnostic tests and may have altered outcomes through intensification of pharmacologic medical therapy.

## Conclusions

In a predominantly intermediate risk group of subjects, vasodilator CMR and CTA findings have excellent agreement. Both tests are accurate for the assessment of obstructive CAD, and non-ischemic vasodilator stress CMR or non-obstructive coronary CTA each confer an excellent prognosis. 

## Abbreviations

CAD: Coronary artery disease
CTA: CT angiography
CMR: Cardiac magnetic resonance imaging
SPECT: Single-photon emission computed tomography
PET: Positron emission tomography
